# Polyandry and the Decrease of a Selfish Genetic Element in a Wild House Mouse Population

**DOI:** 10.1111/j.1558-5646.2011.01336.x

**Published:** 2011-09

**Authors:** Andri Manser, Anna K Lindholm, Barbara König, Homayoun C Bagheri

**Affiliations:** 1 Animal Behaviour/Institute for Evolutionary Biology and Environmental Studies, University of ZurichZurich, Switzerland; 4 Evolutionary Genetics and Theoretical Biology/Institute for Evolutionary Biology and Environmental Studies, University of ZurichZurich, Switzerland

**Keywords:** generation time, intragenomic conflict, *t* haplotype, *t* frequency paradox, overdominance

## Abstract

Despite deleterious effects on individuals, the *t* haplotype is a selfish genetic element present in many house mouse populations. By distorting the transmission ratio, +/*t* males transmit the *t* haplotype to up to 90% of their offspring. However, *t*/*t* individuals perish in utero. Theoretical models based on these properties predict a much higher *t* frequency than observed, leading to the *t* paradox. Here, we use empirical field data and theoretical approaches to investigate whether polyandry is a female counterstrategy against the negative fitness consequences of such distorters. We found a significant decrease of the *t* frequency over a period of 5.5 years that cannot be explained by the effect of transmission ratio distortion and recessive lethals, despite significantly higher life expectancy of +/*t* females compared to +/+ females. We quantified life-history data and homozygous and heterozygous fitness effects. Population subdivision and inbreeding were excluded as evolutionary forces influencing the *t* system. The possible influence of polyandry on the *t* system was then investigated by applying a stochastic model to this situation. Simulations show that polyandry can explain the observed *t* dynamics, making it a biologically plausible explanation for low *t* frequencies in natural populations in general.

In its classical conception, Darwinian evolution by natural selection predicts that genes have to contribute to organismal fitness to be successful. An increasing number of cases are emerging where this paradigm is violated. Selfish genetic elements define such heritable entities. They spread through populations despite being associated with negative fitness consequences for the organism (Burt and Trivers 2006). These stretches of DNA distort Mendelian segregation in their favor (transmission ratio distortion (TRD) or meiotic drive) and thereby gain an advantage over their wild-type variants. Selfish genetic elements fascinate evolutionary biologists because they demonstrate persuasively, that systematic advantages at a gene or gamete level can lead to a distinct disadvantage at a higher level of organization such as the individual or population (Okasha 2006).

## 

### The t haplotype

Since its discovery in 1927 (Dobrovolskaia-Zavadskaia and Kobozieff 1927), the *t* haplotype in house mice *(Mus domesticus)* has been intensively studied and is now the best known example of transmission ratio distorters. Efforts to understand the underlying genetics of this distortion have been quite successful. Heterozygote males produce equal proportions of + and *t* sperm (Silver and Olds-Clarke 1984), but an elaborate poison antidote system exclusively impairs flagellar function of the + sperm (Schimenti 2000; Lyon 2003; Bauer et al. 2005, 2007). This leads to a transmission of *t* gametes that deviates considerably from 50%, and up to as much as 99% (Silver 1993; Lyon 2003). Females, on the other hand, transmit the *t* gametes in the usual Mendelian ratios. The *t* haplotype comprises a complex of linked genes as large as 20 cM (30–40 Mbp), occupying the proximal third of chromosome 17 (Silver 1993). Four major, nonoverlapping inversions block recombination and assure that the complex is transmitted as one intact entity (Artzt et al. 1982). It has been found in all four house mouse subspecies across the world and is thought to have existed in house mouse populations for 1.5 to 2 million years (Silver 1993). As is the case for most known distorter systems, they have negative fitness effects on an individual level. Cases with positive or neutral effects are probably not detected because they will drive to fixation in very short time. Numerous variants of *t* haplotypes have been found. Most t haplotypes carry lethal recessive mutations such that homozygotes for the same variant perish in utero. On the other hand, homozygosity for different, complementing variants (e.g., *t^x^*/*t^y^*) invariably result in male sterility (Klein et al. 1984).

### The t frequency paradox

Despite the solid understanding of the structure and mechanisms, the implications for population dynamics of this genetic polymorphism are still a puzzle (see Ardlie (1998) for a review). In one of the first theoretical approaches to this system more than 50 years ago, Bruck (1957) provided a mathematical model that took into account the antagonism between segregation distortion in males supporting the *t* haplotype frequency, and viability selection at the embryonic stage acting against it. The model assumes random mating and an infinite, unstructured population. Under these assumptions, the frequency of the *t* haplotype reaches a steady-state equilibrium that is surprisingly high, despite the strong negative fitness consequences for *t*/*t* individuals. With a TRD of 0.9, an equilibrium *t* frequency of 0.33 is predicted, meaning that two-thirds of a population are expected to be +/*t* heterozygotes. This expectation is however in marked contrast to frequencies usually found in wild populations. Various empirical studies measuring the frequency of *t* haplotypes in natural populations of different subspecies around the globe show the same picture: frequencies are on a persistent but low level, with *t* frequencies ranging between 0.05 and 0.15 (Ardlie and Silver 1998; Lenington et al. 1988; Huang et al. 2001; Dod et al. 2003). This discrepancy—often called the *t* frequency paradox—has evoked a large number of theoretical models (both analytical models and stochastic simulations) focusing on various forces that might account for this low frequency in a natural context.

### Population subdivision

Population subdivision and genetic drift were considered as possible factors to reduce *t* haplotype frequency, as they reduce heterozygosity and thereby the frequency of *t* carrying individuals (Lewontin and Dunn 1960; Levin et al. 1969; Petras and Topping 1983; Nunney 1993). It has nevertheless been a matter of debate whether mouse populations are as substructured as assumed by these models. Some studies point out that overall *t* frequencies within a population are rather stable (Lenington et al. 1988), whereas empirical data indicate that population size has an influence on *t* allele frequency, with frequencies decreasing in larger populations (Ardlie and Silver 1998).

### Heterozygote fitness effects

Selection against +/*t* heterozygotes will substantially reduce *t* frequencies (Young 1967; Lewontin 1968; Johnston and Brown 1969; Hartl 1970). Here again, empirical data do not show a clear picture. Besides the expected reduction in litter size after a double +/*t* mating, several studies also showed litter size reductions if only one sex was carrying a *t* (Johnston and Brown 1969; Lenington et al. 1994; Carroll et al. 2004). Viability, on the other hand, has been found to be either higher (Dunn et al. 1958) or lower (Carroll et al. 2004) in +/*t* heterozygotes independent of sex.

### Female choice

In a series of studies, Lenington and collaborators found evidence for sexual selection against *t* carrying males via female preferences (Lenington and Egid 1985; Lenington et al. 1994). Using a Y-maze apparatus, they showed repeatedly that +/*t* females spend significantly more time near +/+ male-derived odor cues when given the choice between +/+ and +/*t*. This preference is adaptive in light of the reduction in litter size that would result from a mating with a +/*t* male. However, the question whether these odor preferences reflect actual mating preference is unresolved. Furthermore, male dominance status seems to be an even more important predictor for female olfactory preference (Coopersmith and Lenington 1992), although evidence of the effect of *t* on male dominance is contradictory (Lenington et al. 1996; Carroll et al. 2004).

### Genetic modifiers

Theoretical work predicts the evolution of genetic modifiers suppressing segregation distortion (Charlesworth and Hartl 1978). Such modifiers have been found in various other distorter systems (Hiraizumi and Thomas 1984; Atlan et al. 2003). Surprisingly, they appear to be rare for *t* haplotypes of natural house mouse populations (Ardlie and Silver 1996), although there are reports on suppressors from laboratory mice (Bennett et al. 1983; Gummere et al. 1986).

### Polyandry

Even though a reduction of the segregation ratio due to modifiers seems to be nearly absent in wild populations, there still remains the possibility of a reduction of TRD by other means, such as polyandry. Haig and Bergstrom (1995) proposed the idea that females could avoid individual fitness reduction due to distortion by systematic multiple mating. The hypothesis of polyandry as a general counterstrategy against distorters is based on the observation that gametes carrying the *t* haplotype are “by definition” strong intraejaculate competitors, but do comparatively poorly in competition with other ejaculates (Zeh and Zeh 1996; Zeh and Zeh 1997; Price and Wedell 2008). In comparison to previously described precopulatory mating preferences, this mechanism would not even require the female ability to distinguish between the two different genotypes. Hence, polyandry is a simpler and potentially more robust strategy to counter the spread of *t* haplotypes. A series of empirical studies on several species, predominantly on the genus *Drosophila*, support this idea. Both fertility reductions and reduced competitive ability of sperm from males carrying selfish genetic elements have been found in various systems (see Price and Wedell (2008) for an overview). In *Drosophila pseudoobscura* the X-linked driver (SR) was found to reduce sperm competitive ability (Price et al. 2008a) and females evolved increased remating rates in the presence of the distorter (Price et al. 2008b). Fertility reduction and negative effects on sperm competitive abilities were also found in the SR system and *Wolbachia* of *Drosophila simulans* (Snook et al. 2000; Atlan et al. 2004; Champion de Crespigny and Wedell 2006).

This hypothesis appears to be quite promising for the house mouse case, because recent studies indicate that female house mice are indeed actively polyandrous both in the wild (Dean et al. 2006) and in an experimental context (Rolland et al. 2003). Polyandry provides opportunity for postcopulatory selection processes such as sperm competition and cryptic female choice. It has already been shown that polyandry increases offspring postbirth survival (Firman and Simmons 2008c) and facilitates inbreeding avoidance (Firman and Simmons 2008b). As mentioned above, evidence for negative effects of the *t* on male fertility are convincing: apart from *t*/*t* sterility (in the case of complementing *t* variants), reduced fertility (of about 20%) has invariably been reported in +/*t* heterozygous males (Johnston and Brown 1969; Lenington et al. 1994; Carroll et al. 2004). However, empirical data on sperm competitive abilities in relation to the *t* haplotype are still scarce. One study looking at paternities in a wild population yielded a mean fraction of 0.17 +/*t* among litters involving both +/*t* and +/+ fathers (based on three litters, Ardlie and Silver (1996)), another study using controlled sperm mixing experiments obtained a +/*t* proportion of 0.22 (based on eight litters, Olds-Clarke and Peitz (1986)). In the study of Carroll et al. (2004) on seminatural enclosure populations, the *t* was transmitted to 36% of the offspring.

### Aims

The present study focuses on both empirical and theoretical approaches to understanding the dynamics of the *t* haplotype on a specific wild house mouse population. First, we provide a theoretical model looking at possible impacts of polyandry on *t* haplotype frequency. To our knowledge, no theoretical model has investigated this for *t* haplotypes. However, the evidence in the previous section make polyandry a promising evolutionary force to explain low *t* frequencies in natural populations. Second, extensive data collection in a free-living population of house mice over a time period of more than five years allows us to estimate many parameters likely to affect the *t* frequency. In addition to well-described parameters such as distortion level and homozygous fitness effects, we were able to obtain reliable estimates on heterozygous fitness effects, the degree of inbreeding, as well as *t* frequency dynamics. Reliable estimates of these have not been available for natural house mouse populations so far (Burt and Trivers 2006). In addition, we were able to describe a series of general life-history parameters (generation time, life expectancy, net reproductive rate) as yet largely unknown for wild house mice. In a third step, the theoretical model and the empirical data were compared. We ran computer simulations using the parameters estimated from our population to predict the *t* frequency dynamics. With the present approach, we are able to test specific models with parameters directly estimated from the population of interest.

## Materials and Methods

### THE MODEL

A classical Fisher–Wright population with infinite population size and no mutation is assumed. For the purposes of the model, the whole *t* haplotype is simplified to a single locus with two alleles—the wild-type allele + and the distorter allele *t*. Using *i*= 1 for females and *i*= 2 for males, the variables *p*_+(*i*)_ and *p*_*t*(*i*)_ for each sex denote the frequency of alleles + and *t*, respectively. Similarly, for each sex *i*, the variables *P*_++(*i*)_, *P*_+*t*(*i*),_ and *P*_*tt*(*i*)_ describe the frequency of the genotypes +/+, +/*t*, and *t*/*t*, respectively. For example *P*_++(1)_ would represent the genotype frequency of female homozygote wild-types. *t*/*t* homozygotes are inviable and it simply holds that 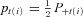 and *P*_++(*i*)_= 1 −*P*_+*t*(*i*)_. This also explains why *p*_*t*(*i*)_ can never exceed 0.5, the case in which all members of the population are heterozygotes. The sex-independent *t* allele frequency *p_t_* can be expressed as *p_t_*=*f*_1_*p*_*t*(1)_+*f*_2_*p*_*t*(2)_, where *f*_1_ and *f*_2_ are relative frequencies of females and males in the population (*f*_1_+*f*_2_= 1).

#### 

##### The Life Cycle

To determine the allele frequencies *p*′_*t*(*i*)_ in the next, nonoverlapping generation, the following life cycle was used.

1. First, adult individuals of the present generation mate with each other. Mating probabilities are based on the genotype frequencies, which means that all matings are random. A certain fraction of the females 0 ≤ψ≤ 1 is assumed to mate twice, whereas the rest of the female population 1 −ψ mate with one randomly encountered male. ψ is independent of female genotype. It is also assumed that matings are not male limited, that is, the number of males does not influence the mating frequencies. The four monotypic matings (two female genotypes can each mate with two male genotypes) and six additional polyandrous matings sum to 10 possible mating combinations (see Table 2). Mating frequencies, expressed as probabilities, can be calculated by multiplying the parental genotype frequencies. For example, the frequency of single matings between a female of genotype *a* and a male of genotype *b* can be expressed as
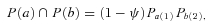
(1)where *a*, *b*∈{++, +*t* }.
  The frequency for a multiple mating involving the ordered male genotypes *b*_1_ and *b*_2_ are given by
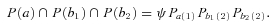
(2)Note that equations (1) and (2) determine the probability for all possible matings, but they do not determine the outcome of the matings. This is done in the next section.
2. After mating, the proportion of the different zygotes of the different crosses and double-crosses is calculated. We distinguish between within and between ejaculate effects.
  *Within ejaculate effects.* Because meiotic drive changes the proportion of functional gametes in heterozygous males, the expected zygotes of the affected crosses deviate from Mendelian predictions. Segregation ratio (proportion of *t* gametes in the functional male gamete pool) in males is characterized by the variable 0 ≤τ≤ 1.
  *Between ejaculates effects.* If a female mates with more than one male, both “quantity” and “quality” of the sperm are assumed to determine the fertilization success of the involved males (gamete fitness). The mating order of the males shall not have an influence on fertilization success. Quantity reduction, represented by the coefficient ν, results from the reduction of functional gametes through ratio distortion τ. Under the assumption that every male produces the same amount of sperm (functional and dysfunctional) per ejaculate, the *fraction* of functional sperm in a given ejaculate is dependent on TRD because the latter operates on rendering + sperm dysfunctional. In +/+ males that do not exhibit TRD, no sperm is lost and thus ν_++_= 1. In +/*t* males, ν is dependent on the level of τ and can be expressed by ν_+*t*_(τ) = (1 + |2τ− 1|)^−1^. The higher the deviation from τ= 0.5, the bigger the individual males' loss in functional sperm. For τ > 0.5, it can also be described as ν_+*t*_(τ) = 1/2τ. Because this expression summarizes the relevant cases for the *t* haplotype and is mathematically easier to handle, it will be used here.
  Differences in quality between the two males ejaculates are modeled with the parameter *c*, which describes the relative disadvantage of the remaining heterozygote derived sperm. Hence, relative competitiveness of +/+ male sperm is *w*_++_= 1, relative competitiveness of +/*t* male sperm *w*_+*t*_= 1 −*c*.
  Given two males of genotype *b*_1_, *b*_2_∈{++, +*t* }, the probability 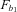 of fertilization of a given egg by the male of genotype *b*_1_ is dependent on both gamete fitness components *w* and ν and can be expressed by
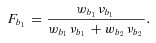
(3)
  If both males have the same genotype (e.g., *b*_1_=*b*_2_=‘+*t*’), then 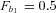. Note that 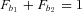. Knowing this, the proportions of zygote genotypes produced by each mating cross *P^z^_a_* can be determined (see Table 1 for one illustrative example and Table 2 for an overview over all possible mating crosses). The zygote frequencies of the total population 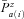 of the genotypes *a*∈{++, +*t*, *tt* } are then given by the sum of the outcomes of each individual mating cross *P^z^_a_* weighed by the frequency of the respective mating cross derived in equations (1) and (2).
3. In the next step, viability selection occurs. The different genotypes are given different probabilities to establish themselves as adults in the population. Estimates from our wild population suggest that these probabilities can be sex dependent and eventually lead to sexually antagonistic effects. Relative viability differences between +/+ and +/*t* individuals will be characterized by *s_i_*, where *i* again defines sex. Overdominance is indicated by *s_i_* < 0 and underdominance by *s_i_* > 0. Thus, relative viabilities for +/+ genotypes are *w*_++(*i*)_= 1 and *w*_+*t*(*i*)_= 1 −*s_i_* for +/*t* genotypes, respectively. Because the *t* haplotypes of our study population carry an embryonic lethal (A. K. Lindholm, unpubl. data), viability of homozygote *t* carriers is set to be *w*_*tt*(*i*)_= 0. Adult genotype frequencies for any genotype *a*∈{++, +*t* } after viability selection are given by
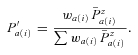
(4)
  We use a scenario in which ψ= 0, *c*= 0, and *s_i_*= 0 as a null model. This is equivalent to the model by Bruck (1957), and is a case of dominance of the wild-type allele, because both +/+ and +/*t* have the same fitness, whereas *t*/*t* are lethal.

**Table 1:** One example showing how to calculate the zygote frequencies 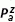 of genotypes *a*∈{++, +*t*, *tt*} for the multiple cross involving a female of genotype *a*=+*t* and males of genotype *b*_1_=++ and *b*_2_=+*t*. 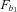 and 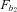 are derived from equation (3). The zygote frequencies for all other crosses can be calculated likewise

		**Male***b*_1_	**Male***b*_2_		
				
	Gametes	+	+	*t*	Total
**Female***a*	+	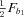	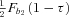	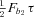	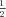
	*t*	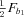	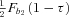	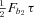	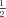
	Total	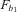	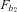		1
**Resulting zygote frequencies** (using eq. 3)					
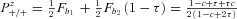					
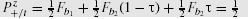					
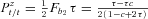					

**Table 2:** The different possible single (female of genotype *a* mating with a male of genotype *b*) and multiple crosses (female of genotype *a* mating with males of genotypes *b*_1_, *b*_2_) in the polyandry model, including their resulting zygote proportions (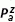 for *a*∈{++, +*t*, *tt*}). Expected paternity shares at birth (correcting for *t*/*t* lethality) of the first male *b*_1_, and the second male *b*_2_, are given in the cases of multiple crosses

		Zygote frequencies per mating cross			Paternity shares at birth	
			
		*P^z^*_++_	*P^z^*_+*t*_	*P^z^_tt_*	*b*_1_	*b*_2_
	+/t *+/t	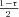	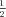	τ/2		
Single matings	+/+*+/t	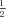	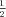	0		
male *b*× female *a*	+/t *+/+	1 −τ	τ	0		
	+/+*+/+	1	0	0		
	(+/t, +/t) *+/t	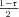	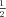	τ/2	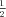	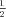
	(+/+, +/+) *+/t	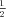	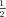	0	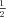	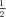
Multiple matings	(+/+, +/t) *+/t	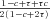	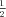	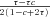	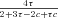	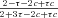
(male *b*_1_, male *b*_2_) × female *a*	(+/+, +/+) *+/+	1	0	0	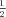	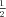
	(+/t, +/t) *+/+	1 −τ	τ	0	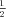	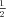
	(+/+, +/t) *+/+	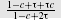	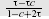	0	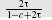	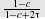

### THE STUDY POPULATION

All the data used in the present study originate from a free-living population inhabiting a barn near Zurich. The population was established in 2002 from 12 founder individuals (originating from two different capture sites close-by). On an area of 72 {m^2^, animals were given breeding opportunities (40 nest boxes) and ad libitum food and water (around eight drinking and feeding sites). Branches and shelves allowed mice to establish several territories in the building and freely enter and leave the barn through numerous openings. The population has been intensively monitored from inception for births and deaths, and tissue samples for genetical analyses have been taken from each pup and adult, as well as from individuals found dead. For the period between 2003 and summer 2008 used for the present study, 2177 pups were sampled at an age of 13–15 days. We found a rate of about 5% unsampled individuals among all captured adults during this study period. We thus sampled at least 95% of all pups born in the population.

### GENOTYPING

To identify the *t* haplotype, the *Hba-ps4* locus — a marker containing a 16-bp *t* haplotype-specific insertion (Hammer et al. 1989) — was amplified and scored. Sexing of pups was performed by amplification of three different Y-specific microsatellite markers (Y8, Y12, and Y21; Hardouin et al. (2010) and M. Teschke, pers. comm.). Samples in which no Y marker amplified were scored as females (A. K. Lindholm, unpubl. data). Identification of dead and adult individuals was also carried out by matching multi-locus genotypes of the dead or adult individuals with those of the sampled pups at 21 unlinked microsatellite loci, allowing one mismatch (A. K. Lindholm, unpubl. data). As pups were sampled at an age of 13 days, birth date could be calculated. Using the date on which the dead body was found as date of death, longevity could be determined.

## Results

### PREDICTIONS BASED ON DETERMINISTIC MODEL

**Figure 1:**
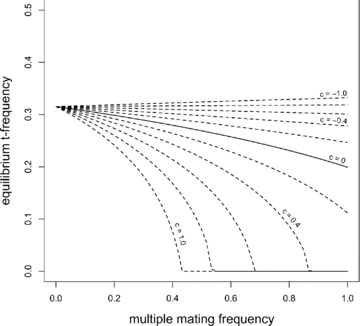
Equilibrium *t* frequency 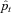 dependent on multiple mating frequency (ψ) for different levels of *t* sperm competitiveness −1 ≤*c*≤ 1 and τ= 0.90. The solid line represents the case where heterozygote males only suffer from quantitative sperm disadvantages resulting directly from segregation ratio distortion (*c*= 0).

To investigate the potential impact of polyandry on the *t* frequency *p_t_*, the life cycle described by equations (1–4) was repeated for 1000 generations. Independent of the initial parameter settings, *p_t_* reached a steady-state equilibrium 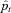 after only a few generations. Figure 1 shows 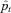 dependent on multiple mating frequency ψ and heterozygote relative sperm competitiveness *c*. Segregation distortion was set to the level observed in our population at τ= 0.90 (see Supporting infor). If +/*t* males have only a quantitative sperm competition disadvantage (lower viable sperm count) compared to +/+ males (*c*= 0, solid line in Fig. 1), polyandry does not have strong effects on *t* frequency. When all females mate multiply (ψ= 1), the proportion of +/*t* individuals in a population is reduced by about 20% compared to the case where only single matings occur (ψ= 0). Frequency of matings involving a +/*t* and a +/+ male—the cases where sperm competition disadvantage can play a role at all—is obviously too rare to produce substantial effects on 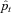. This changes however, if disadvantages in sperm quality are included in the quantitative sperm competition disadvantage of *t* carrying males (*c* > 0). Higher disadvantages in +/*t* sperm quality lead to lower equilibrium *t* frequencies. Depending on the level of *c*, effects on *t* frequency can be quite strong, especially when accompanied by high multiple mating rates.

### PREDICTIONS BASED ON STOCHASTIC MODEL AND COMPARISON TO WILD HOUSE MOUSE POPULATION

#### 

##### Empirical observations

**Figure 2:**
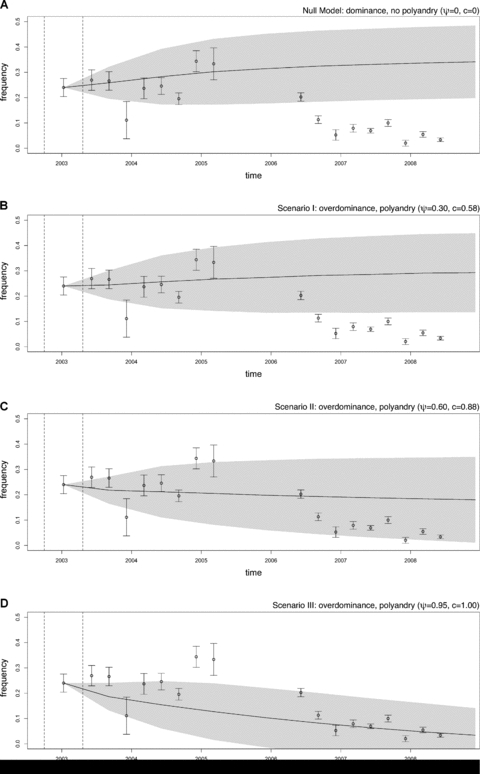
Observations from the wild population and simulation predictions from October 2002 until June 2008. The first dot within vertical dashed lines represents the initial *t* frequency among the first 50 adults ± SE. The following dots represent the *t* frequency among the pups born in the given 3 month time interval ± SE (*n_tot_*= 2177). Solid lines show model predictions with 95%-confidence intervals (shadows) for (A) the null model (dominance and no polyandry) and (B)–(D) scenarios I–III (in Table 3, overdominance and different levels of polyandry). [Correction added June 1, 2011 after Online publication: Figure 2 legend updated to reflect black-and-white publication.]

The model was applied to an intensively studied, free-living house mouse population near Zurich. A lethal version of the *t* haplotype was present in this population since its establishment. Figure 2A shows the frequency of the *t* over a 5.5-year time period (2003 until summer 2008) among 2177 pups. Although fluctuating substantially, *t* frequency decreased significantly over these five years (GLM on proportions using a binomial error structure and a logit link function using time as a continuous factor (in years); residual deviance: χ^2^_17_= 79.53, *P* < 0.001).

##### The t paradox

**Figure 3:**
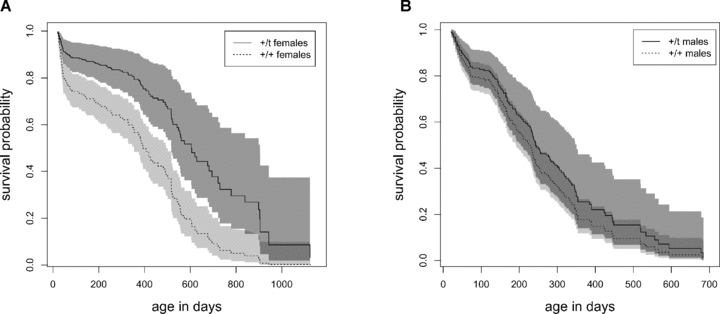
Empirically estimated survival functions for (A) females and (B) males of different genetic backgrounds based on a Cox model. Each estimate is accompanied by a pointwise 95% confidence envelope.

This decrease is surprising given the substantial advantage for the *t* haplotype through segregation ratio distortion. For the levels of segregation ratio distortion found in the population (τ= 0.90, see Supporting information for more details), Bruck (1957) predicts an equilibrium *t* frequency 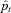 as high as 0.32 (see Fig. 2A). In addition, we found that *t* heterozygote females have a significantly longer life expectancy than homozygote wild-type females (Cox proportional hazard model: *n*= 174, exp(β)=2.46, *P* < 0.01, see Figure 3 and Supporting information for more details). Male survival on the other hand was not dependent on the *t* haplotype (Cox proportional hazard model: *n*= 185, exp(β)=1.26, *P*= 0.31). The higher survival probabilities of female heterozygotes hence leads to overdominance (as compared to the Null model, which is a case of dominance). We used data from 21 unlinked microsatellite markers to examine inbreeding. Inbreeding levels did not change throughout the whole observation period. A linear regression analysis showed that the *F_IS_* (deviations in heterozygosity from Hardy–Weinberg predictions) did not significantly change over time (Regression coefficients: α= 0.0259, β=−0.0065, *n*= 147, *P*= 0.07, *r*= 0.023, see Fig. S1).

##### Polyandry

Based on paternity analysis, we find a proportion of litters sired by more than one male (multiple paternity rate) of around 0.3 (Camani 2005). Competitive ability *c* of +/*t* derived sperm was estimated from laboratory experiments with wild mice (A. Manser, unpubl. data). Using an elaborate choice device that allowed females free physical access to both a +/+ and a +/*t* male without male interference, we found a proportion of 0.19 (95% CI using a binomial error distribution: [0.08, 0.35]) sired by the +/*t* male among four multiply sired litters. The experimental setup did not control for mating order. This would correspond to *c*= 0.58 (following Table 2). We are, however, facing a fundamental problem. Multiple paternity rate and sperm competitiveness of *c*= 0.58 mark only the “minimal” amount of sperm competition possible in our system. Cases of multiple matings with only one successful competitor do not lead to multiple paternity and are therefore missed by our analysis. The amount of such cases highly depends on sperm competitiveness *c*, the litter size λ as well as *t* frequency *p_t_* in the population. But we cannot estimate both *c* and ψ only knowing multiple paternity rate. We are left with an underdefined system that forces us to make assumptions.

Scenario I: This most conservative scenario assumes that all multiple matings resulted in multiple paternities. This leaves us with a multiple mating frequency of ψ= 0.3 and a *t* sperm competitiveness of *c*= 0.58.
Scenario II: In this intermediate scenario, multiple mating frequency is assumed to be slightly higher. ψ= 0.6 is a value that is within the range of what has previously been described for wild house mouse populations (Dean et al. 2006). By making use of our model (with known litter size λ and mean *t* frequency 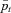) we find that *c*= 0.88 is needed to explain a gap between multiple paternity and multiple mating of this order.
Scenario III: The third scenario assumes an almost maximal multiple mating rate. It is based on the only study that actually looked at the mating behavior of females in an experimental context (Rolland et al. 2003). Given the choice between two different males, they found that 20 of 21 females (95%) mated with both of the males. Therefore, this scenario assumes ψ= 0.95 and *c*= 1.

##### Stochastic model

To determine if polyandry can explain this dramatic decrease in frequency, we ran simulations based on parameters estimated from the population (see Table 3 and Supporting information for a more detailed description). The deterministic, panmictic model described before was slightly modified. The deterministic nature of the model assuming infinite population size was now replaced by a stochastic simulation sampling randomly through the different life cycle stages based on effective population size and mean litter size. Life-history data from the populations allowed us to calculate an average time to reproduction (generation time) of 9 months (see Supporting information). This estimate was used to fit the simulation assuming nonoverlapping generations to the study population, where generations overlap. To get reliable estimates on model predictions and its confidence intervals, the 5.5-year observational period was simulated 10,000 times for each scenario. Initial frequency was set to the *t* frequency among the first 50 adults (equal to *N_e_*, shown in red in Fig. 2, separated by a dotted line) which was *p_t_*= 0.24.

**Table 3:** Parameter estimates used in this study. Roman numerals refer to the three scenarios tested. Estimation of the lower part is shown in the Suppo

	Definitions	Estimates
ψ	Frequency of females mating twice	I: 0.30
		II: 0.60
		III: 0.95
*c*	Relative disadvantage of +/*t* derived sperm	I: 0.58
		II: 0.88
		III: 1.00
λ	Average litter size at birth	5.47
γ	Generation time	9 months
*N_e_*	Effective population size	50
τ	Segregation ratio distortion	0.90
*s*_1_	Difference in survival between +/+ females and +/*t* females	−0.30
*s*_2_	Difference in survival between +/+ males and +/*t* males	0.00

Figure 2A shows the stochastic model predictions for the null model (following Bruck (1957)), which assumes no polyandry, equal fitness between +/+ and +/*t* individuals, and homozygote *t*/*t* lethality (dominance). Figure 2B–D shows the three different polyandry scenarios that include female overdominance and sperm competition. Scenarios 2 and 3 with high polyandry fit the data considerably better than the null model. Notice that—in contrast to scenario 2—the deterministic equilibrium prediction for scenario 3 is the extinction of the *t*. However, more generations than used in the present simulation are on average needed to reach extinction.

### EFFECTS ON MEAN POPULATION FITNESS

**Figure 4:**
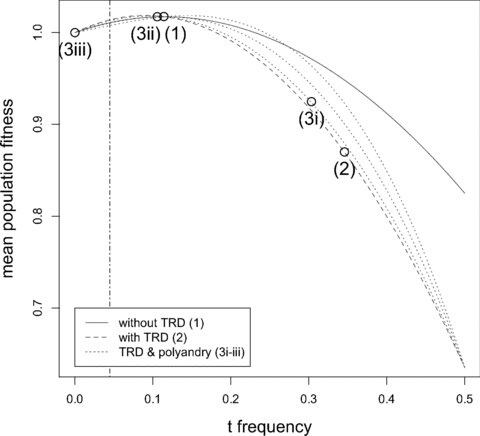
Mean population fitness for the offspring generation as a function of the *p_t_* of the parental generation (assuming 1:1 male to female ratio) and their associated equilibrium frequencies (dots) for no TRD and polyandry (solid gray line and point (1)), TRD without polyandry (dashed gray line and point (2)), and the complete models with TRD and polyandry (dotted gray lines and point (3i–iii) corresponding to scenarios I–III). The vertical line with dash-points corresponds to observed *t* frequency in the population in summer 2008 (GLM prediction: *p_t_*= 0.045).

Examining mean fitness of the overall population here reveals interesting effects of the *t* haplotype. As is the case for all selfish genetic elements (Burt and Trivers 2006), the *t* haplotype elicits conflict between different levels of selection (allelic propagation versus population fitness). This conflict can be revealed when looking at the mean population fitness and its related gene frequencies. In the case of overdominance, mean population fitness is generally optimized by maximizing the number of heterozygotes in a population (Wright 1929). However, in the presence of distorters, the expectation that Mendelian segregation and random mating lead to gene frequency changes that increase mean population fitness (Wright 1929) no longer holds. By increasing their own frequency, *t* haplotypes drag the population frequency away from the optimum. This is shown in Figure 4 for our specific case: in accordance with Wright, without segregation distortion (solid line) an equilibrium frequency that optimizes population fitness is reached (point [1]). As soon as we add meiotic drive to the system (dashed line), mean population fitness at equilibrium is far from the optimum (point [2]), manifesting the conflict between gene and population level fitness. Dependent on the scenario, these population fitness losses are recovered by the process of sperm competition. Whereas scenario 2 has an equilibrium *t* frequency that nearly corresponds to the optimum of population fitness, the deterministic model predicts extinction of the *t* and suboptimal population fitness in scenario 3. The observed *t* frequency at the end of the observation period (shown as vertical dash-dotted line) falls between maximum population fitness and extinction of the *t* haplotype.

## Discussion

### 

#### t dynamics

One of the main findings of this study is the significant decrease in *t* frequency in a wild population over a time period of five years. It is remarkable how strong the dynamics of the *t* haplotype in our study population closely resemble the patterns observed in other studies that examined *t* frequencies in a natural or seminatural context. Both Ardlie and Silver (1998) and Carroll et al. (2004) find low frequencies in their study populations of house mice, the latter report a similar decrease in frequency over time in their enclosure populations. Even in a different subspecies *(Mus castaneus)* in Taiwan, *t* frequencies are found at persistent but low frequencies (Huang et al. 2001). These striking parallels among studies measuring *t* frequencies in different geographic and phylogenetic contexts suggest similar general mechanisms. It also confirms our assumption that if we are able to unravel the factors determining the *t* dynamics in our specific population, these findings have implications for house mouse populations in general.

Parallels to other studies are not only restricted to *t* frequency dynamics, as most other parameter estimates confirm what has previously been found. TRD of 0.90 is a level typically observed in other natural and semi-natural populations (Ardlie and Silver 1996; Huang et al. 2001; Carroll et al. 2004), and provides no indication of a reduction of distortion level by modifiers (Ardlie 1998). The estimates concerning polyandry in our study population are also consistent with previous investigations. A marked multiple paternity rate of about 30% (Camani 2005) provides further support that house mice mating systems are highly polygynandrous. This estimate is further in high accordance with recent work, that found multiple paternity rates of 23% and 26% (Dean et al. 2006; Firman and Simmons 2008a).

Competitiveness of the +/*t* derived sperm *c* is clearly the model parameter with the weakest support, both in our study and in the literature. Two other studies measured +/*t* male paternity shares of 0.17 and 0.22 (Olds-Clarke and Peitz 1986; Ardlie and Silver 1996), proportions that are comparable to what has been found here (0.19). They contradict however slightly with Carroll et al. (2004) who found a paternity share for +/*t* males of 0.36, a value that would suggest no “qualitative” differences in sperm (for *c*= 0 and τ= 0.90 our model predicts a +/*t* heterozygotes paternity share of *F*_+*t*_= 0.28). Better estimates for this key parameter of the model are clearly needed to further support the polyandry hypothesis.

#### Female fitness advantage

+/*t* females in our population have a longer life expectancy, which is in marked contrast to what has previously been reported. An early study found similar fitness advantages in both sexes (Dunn et al. 1958), but most recent publications show a different picture. In a detailed study on the fitness consequences of the *t*, Carroll et al. (2004) find fitness disadvantages for *t* carriers both in males and females (both in survivorship and reproductive output). They argue further that artificially high levels of competition in their enclosure populations might amplify these differences. Indeed, we find indicators of high competition such as wounded adult males and females, and dead pups inside and outside nest boxes (presumably killed by conspecifics) in our study population—apparently not only among males, but surprisingly also among females. The fact that in contrast to the study of Carroll et al. (2004), individuals always had the opportunity to leave the population, makes this even more surprising. Reproductive skew turned out to be strong both in males and females (A. K. Lindholm, unpubl. data). Furthermore, more pups are produced each year than are able to recruit into the population (see Supporting information). However, we have no information about what happens to mice that disperse out of the population. Our longevity estimates are based on nondispersing mice out of the barn, thus our result of genotype-dependent survival makes the assumption that there is no differential emigration. Even if our survival data are confounded with migration, it nevertheless seemed to be appropriate to use the longevity estimates for the model, because they provide reliable information about the probability of an individual to establish in the population of interest.

#### The conflict between different levels of selection

The implications of the combination of fitness effects between individual and mean population fitness are intriguing. Weak overall overdominance results if female positive and male neutral effects are merged together (see Fig. 4). It follows from this that maximum population fitness is reached if a considerable number of *t* haplotypes are still present in the population. This finding is counterintuitive, because distorter systems are usually characterized as “genomic parasites.” In this case however, a certain amount of parasites is beneficial to the collective. This would suggest that there is an intermediate optimal rate of polyandry, as Arnqvist and Nilsson (2000) suggest in a meta-analysis on insects. Nevertheless, negative effects are still severe if two parasites occur in the same individual (*t*/*t* homozygosity). Meiotic drive makes these cases more frequent. The relation between equilibrium gene frequency and population fitness (Fig. 4) illustrates how nonrandom segregation can lead to outcomes that are no longer maximizing individual or population fitness. Sexual selection in the form of polyandry and sperm competition is a possible mechanism to recover losses in individual and population fitness by reducing the number of *t*/*t* zygotes produced. Hence, multiple mating can be seen as an adaptive strategy for the organism to increase individual fitness. The question however, if an internal *t* frequency equilibrium optimizing mean population fitness is reached, depends on the scenario. The observed *t* frequency dynamics do not offer a conclusive answer to this question, because *t* frequency at the end of the 5.5-year period falls between the population fitness optimum and *t* extinction.

The conflict between levels of selection can also be seen as a conflict among the genes. Nonlinked genes can only spread through populations when they increase individual fitness (as is the case if mean population fitness is maximized). In this respect, polyandry would represent an indirect solution to intragenomic conflict: by building up organisms that pursue a polyandrous mating strategy, unlinked genes are able to prevent *t* haplotypes from reaching frequencies that are too high. However, the overall overdominance effect is only weak and appears to be rather specific for the observed population. Thus, maximization of population fitness may not provide a general answer to the question of why *t* haplotypes generally occur at small but stable frequencies.

#### The model predictions

Our specific case study provides a further example that the null model provided by Bruck (1957)—even when allowing for drift—is not sufficient to explain the naturally observed frequencies. In contrast to the null model, our model shows that polyandry in the context of a meiotic drive system is a potential mechanism to explain the low *t* frequency paradox.

There are plenty of reasons to assume that sperm competition levels are substantially higher than assumed in scenario I. First, the assumption of a maximal number of two matings per female is clearly a simplification. Rolland et al. (2003), for example, showed that females can regularly change mating partners during one estrous cycle (13 out of 21 females received three ejaculations from two potential mating partners during one estrous cycle). The probability *p* of having *at least* one *t* carrying male among all males mated increases with every mating partner. If *k* is the number of partners per estrous cycle and *P*_+*t*(2)_ the proportion of heterozygote males in a population, this probability increases with *k* given *p*= (1 −*P*_+*t*(2)_)^*k*^. This effect would increase the number of matings where the +/+ sperm competition advantage plays a role. Second, we only looked at competitiveness effects dependent on the donor's genotype. This is of course a simplification, because other factors such as mating order usually have a major impact on reproductive success. The integration of such factors would not change our predictions of *t* frequencies (assuming that the mating order remains random). However, it would strongly increase the discrepancy between multiple matings and the actually measured multiple paternities. This stresses once more the urgency to estimate the specific factors determining male reproductive success in sperm competition in the future.

Obviously, there is still the possibility that the forces included in the present model are insufficient to explain the observed *t* dynamics. However, most of the potential factors that have been proposed in the last 50 years have been considered here. Population substructure appears rather unlikely given that migration within the barn is clearly too high to result in different demes. Even if there was a certain amount of substructure in the barn, Levin et al. (1969) showed that only small migration rates are needed to counterbalance the loss of *t* haplotypes by genetic drift as described in Lewontin and Dunn (1960). Inbreeding (Petras and Topping 1983) was excluded here as a possible factor (see Fig. S4). Migration of *t* carriers into the population is unlikely to affect *t* dynamics: We only found 5% unmarked adults (see Material and Methods) and genetic analyses did not identify immigrants among them (A. K. Lindholm, unpubl. data). There is also the possibility that precopulatory female mating bias interacts with the here described postcopulatory effects of sperm competition. At first sight, one might think that these two hypotheses could be exclusive. Why should females mate with multiple partners if they can recognize and discriminate between the different genotypes (as indeed suggested by Coopersmith and Lenington (1992))? However, female mating decisions might not always be as free and unconstrained as assumed. If the dominant male of their territory is a *t* carrier, females may not have any choice but to mate with him or risk infanticide (Perrigo et al. 1991). Results by Coopersmith and Lenington (1992) indicate that male dominance status is rated higher by females than genetic background. If one would assume that this preference is adaptive, this result would indicate that the costs for a female to mate exclusively with a subordinate male are even higher than the costs of mating with a +/*t* male. However, the correlation between male dominance and the *t* genotype is unclear. In arena experiments, +/*t* males tended to dominate +/+ males (Lenington et al. 1996), whereas Carroll et al. (2004) found +/+ males to be dominant in a seminatural context. In any case, an interaction of both multiple mating and precopulatory mate choice could definitely have more profound effects on equilibrium *t* frequency. It would be very interesting to investigate this further. For example, one might expect an increase in polyandry in the case of many *t* carrying males present in a population, as Price et al. (2008b) showed in *D. pseudoobscura*.

#### General implications

Many have wondered why one cannot find modifiers in the genome that fight *t* distortion on a genetic level in naturally occurring house mouse populations (Ardlie 1998). The previously described X-linked *SR* system in *D. pseudoobscura* is one of the other cases where no modifiers have been found so far (Beckenbach 1996). The results presented here and those of Beckenbach (1996) and Ardlie (1998) suggest an alternative scenario: the possibility that there was never a need for such suppressors to evolve, because the *t* haplotype and the *SR* were already facing severe problems to be successful in polygynandrous mating systems. Thus, the mechanisms that such genetic elements use to make them strong intraejaculate competitors (reduced number of + sperm) can at the same time make them vulnerable to sperm competition, and hence, multiple mating. Individual behavior in the form of female mating decisions would in this case improve individual fitness (to the disadvantage of the *t*). Alternatively, the genes determining multiple mating could be regarded as another class of genetic modifiers of segregation distortion.

The results presented here demonstrate that polyandry is a biologically plausible explanation for the low *t* frequency paradox.

## Supporting Information

The following supporting information is available for this article:

**Text S1.** Parameter Estimation

**Figure S1.** Inbreeding.

**Figure S2.** Pup survival.

**Figure S3.** Overall survival and fertility curves.

## References

[b1] Ardlie K (1998). Putting the brake on drive: meiotic drive of *t* haplotypes in natural populations of mice. Trends Genet.

[b2] Ardlie K, Silver L (1996). Low frequency of mouse *t* haplotypes in wild populations is not explained by modifiers of meiotic drive. Genetics.

[b3] Ardlie K, Silver L (1998). Low frequency of *t* haplotypes in natural populations of house mice *(Mus musculus domesticus)*. Evolution.

[b4] Arnqvist G, Nilsson T (2000). The evolution of polyandry: multiple mating and female fitness in insects. Anim. Behav.

[b5] Artzt K, McCormick P, Bennett D (1982). Gene mapping within the *T*/*t* complex of the mouse. I: *t*-lethal genes are nonallelic. Cell.

[b6] Atlan A, Capillon C, Derome N, Couvet D, Montchamp-Moreau C (2003). The evolution of autosomal suppressors of sex-ratio drive in *Drosophila simulans*. Genetica.

[b7] Atlan A, Joly D, Capillon C, Montchamp-Moreau C (2004). Sex-ratio distorter of *Drosophila simulans* reduces male productivity and sperm competition ability. J. Evol. Biol.

[b8] Bauer H, Véron N, Willert J, Herrmann B (2007). The *t*-complex-encoded guanine nucleotide exchange factor *Fgd2* reveals that two opposing signaling pathways promote transmission ratio distortion in the mouse. Genes Develop.

[b9] Bauer H, Willert J, Koschorz B, Herrmann B (2005). The *t* complex-encoded GTPase-activating protein *Tagap1* acts as a transmission ratio distorter in mice. Na. Genet.

[b10] Beckenbach A (1996). Selection and the ‘sex-ratio’ polymorphism in natural populations of *Drosophila pseudoobscura*. Evolution.

[b11] Bennett D, Alton A, Artzt K (1983). Genetic analysis of transmission ratio distortion by *t*-haplotypes in the mouse. Genet. Res.

[b12] Bruck D (1957). Male segregation ratio advantage as a factor in maintaining lethal alleles in wild populations of house mice. Proc. Natl. Acad. Sci. USA.

[b13] Burt A, Trivers R (2006). Genes in conflict: the biology of selfish genetic elements.

[b14] Camani M (2005). Genetic evidence of multiple paternity in a wild house mouse population.

[b15] Carroll L, Meagher S, Morrison L, Penn D, Potts W (2004). Fitness effects of a selfish gene (the *Mus t* complex) are revealed in an ecological context. Evolution.

[b16] Charlesworth B, Hartl D (1978). Population dynamics of the segregation distorter polymorphism of *Drosophila melanogaster*. Genetics.

[b17] Coopersmith C, Lenington S (1992). Female preferences based on male quality in house mice: interaction between male dominance rank and *t*-complex genotype. Ethology.

[b18] Champion de Crespigny F, Wedell N (2006). *Wolbachia* infection reduces sperm competitive ability in an insect. Proc. R. Soc. Lond. B.

[b19] Dean M, Ardlie K, Nachman M (2006). The frequency of multiple paternity suggests that sperm competition is common in house mice *(Mus domesticus)*. Mol. Ecol..

[b20] Dobrovolskaia-Zavadskaia N, Kobozieff N (1927). Sur la reproduction des souris anoures. Comptes Rendus Séances Société de Biologie et de ses Filiales.

[b21] Dod B, Litel C, Makoundou P, Orth A, Boursot P (2003). Identification and characterization of *t* haplotypes in wild mice populations using molecular markers. Genet. Res..

[b22] Dunn L, Beasley A, Tinker H (1958). Relative fitness of wild house mice heterozygous for a lethal allele. Am. Nat..

[b23] Firman R, Simmons L (2008a). The frequency of multiple paternity predicts variation in testes size among island populations of house mice. J. Evol. Biol..

[b24] Firman R, Simmons L (2008b). Polyandry facilitates postcopulatory inbreeding avoidance in house mice. Evolution.

[b25] Firman R, Simmons L (2008c). Polyandry, sperm competition, and reproductive success in mice. Behav. Ecol..

[b26] Gummere G, McCormick P, Bennett D (1986). The influence of genetic background and the homologous chromosome 17 on *t*-haplotype transmission ratio distortion in mice. Genetics.

[b27] Haig D, Bergstrom C (1995). Multiple mating, sperm competition and meiotic drive. J. Evol. Biol..

[b28] Hammer M, Schimenti J, Silver L (1989). Evolution of mouse chromosome 17 and the origin of inversions associated with *t* haplotypes. Proc. Natl. Acad. Sci. USA.

[b29] Hardouin EA, Chapuis JL, Stevens MI, van Vuuren JB, Quillfeldt P, Scavetta RJ, Teschke M, Tautz D (2010). House mouse colonization patterns on the sub-Antarctic Kerguelen Archipelago suggest singular primary invasions and resilience against re-invasion. BMC Evol. Biol..

[b30] Hartl D (1970). A mathematical model for recessive lethal segregation distorters with differential viabilities in the sexes. Genetics.

[b31] Hiraizumi Y, Thomas A (1984). Suppressor systems of segregation distorter *(SD)* chromosomes in natural populations of *Drosophila melanogaster*. Genetics.

[b32] Huang SW, Ardlie KG, Yu HT (2001). Frequency and distribution of *t*-haplotypes in the Southeast Asian house mouse *(Mus musculus castaneus)* in Taiwan. Mol. Ecol..

[b33] Johnston P, Brown G (1969). A comparison of the relative fitness of genotypes segregating for the *t*^*w*2^ allele in laboratory stock and its possible effect on gene frequency in mouse populations. Am. Nat..

[b34] Klein J, Sipos P, Figueroa F (1984). Polymorphism of *t*-complex genes in European wild mice. Genet. Res..

[b35] Lenington S, Coopersmith C, Erhart M (1994). Female preference and variability among *t*-haplotypes in wild house mice. Am. Nat..

[b36] Lenington S, Drickamer L, Robinson A, Erhart M (1996). Genetic basis for male aggression and survivorship in wild house mice *(Mus domesticus)*. Aggressive Behav..

[b37] Lenington S, Egid K (1985). Female discrimination of male odors correlated with male genotype at the *T* locus: a response to *T*-locus or *H-2*-locus variability?. Behav. Genet..

[b38] Lenington S, Franks P, Williams J (1988). Distribution of t-haplotypes in natural populations of wild house mice. J. Mammal..

[b39] Levin B, Petras M, Rasmussen D (1969). The effect of migration on the maintenance of a lethal polymorphism in the house mouse. Am. Nat..

[b40] Lewontin R (1968). The effect of differential viability on the population dynamics of *t* alleles in the house mouse. Evolution.

[b41] Lewontin R, Dunn L (1960). The evolutionary dynamics of a polymorphism in the house mouse. Genetics.

[b42] Lyon M (2003). Transmission ratio distortion in mice. Annu. Rev. Genet..

[b43] Nunney L (1993). The role of deme size, reproductive patterns, and dispersal in the dynamics of *t*-lethal haplotypes. Evolution.

[b44] Okasha S (2006). Evolution and the levels of selection.

[b45] Olds-Clarke P, Peitz B (1986). Fertility of sperm from *t*/+ mice: evidence that +-bearing sperm are dysfunctional. Genet. Res..

[b46] Perrigo G, Belvin L, Vom Saal F (1991). Individual variation in the neural timing of infanticide and parental behavior in male house mice. Physiol. Behav..

[b47] Petras M, Topping J (1983). The maintenance of polymorphisms at two loci in house mouse *(Mus musculus)* populations. Genome.

[b48] Price T, Bretman A, Avent T, Snook R, Hurst G, Wedell N (2008a). Sex ratio distorter reduces sperm competitive ability in an insect. Evolution.

[b49] Price T, Hodgson D, Lewis Z, Hurst G, Wedell N (2008b). Selfish genetic elements promote polyandry in a fly. Science.

[b50] Price T, Wedell N (2008). Selfish genetic elements and sexual selection: their impact on male fertility. Genetica.

[b51] Rolland C, MacDonald D, Berdoy M (2003). Free female choice in house mice: leaving best for last. Behaviour.

[b52] Schimenti J (2000). Segregation distortion of mouse *t* haplotypes the molecular basis emerges. Trends Genet..

[b53] Silver L (1993). The peculiar journey of a selfish chromosome: mouse *t* haplotypes and meiotic drive. Trends Genet..

[b54] Silver L, Olds-Clarke P (1984). Transmission ratio distortion of mouse *t* haplotypes is not a consequence of wild-type sperm degeneration. Develop. Biol..

[b55] Snook R, Cleland S, Wolfner M, Karr T (2000). Offsetting effects of *Wolbachia* infection and heat shock on sperm production in *drosophila simulans* analyses of fecundity, fertility and accessory gland proteins. Genetics.

[b56] Wright S (1929). Fisher's theory of dominance. Am. Nat..

[b57] Young S (1967). A proposition on the population dynamics of the sterile *t* alleles in the house mouse. Evolution.

[b58] Zeh J, Zeh D (1996). The evolution of polyandry I: intragenomic conflict and genetic incompatibility. Proc. R. Soc. Lond. B.

[b59] Zeh J, Zeh D (1997). The evolution of polyandry II: post-copulatory defences against genetic incompatibility. Proc. R. Soc. Lond. B.

