# Supplementary material for: Polyandry and the Decrease of a Selfish Genetic Element in a Wild House Mouse Population
Source: Evolution. 2011 Sep;65(9):2435–47. doi: 10.1111/j.1558-5646.2011.01336.x (PMC3187861; doi:10.1111/j.1558-5646.2011.01336.x)
Supplement: Supplementary file 1 [file evo0065-2435-SD1.pdf]

Supporting Information

Text S1 — Parameter Estimation

γ, λ — **Generation time, Average litter size at birth (Life history parameters).** To get a reliable estimate for generation time (the average time to reproduction) both overall survival and fertility dependent on age were used (l_x and m_x curves).

Survival. Since genetic sampling is only possible after an age of 13 days, newborns can not be followed individually before the day of sampling and therefore require a separate analysis. Therefore, survival estimation was divided into two parts: pup survival (until an age of 15 days, equivalent to weaning age) and adult survival (from 15 days onwards).

A comparison in litter size between the date of first discovery and the date of genetical sampling (usually around day 13) can be used to get an estimate for pup survival. For a total of 217 litters, information about its size when first found and at day of genetical sampling was available. Litter size decreased significantly with time (linear regression, $n = 434$, $p < 0.001$, $r = 0.07$) with an average loss of 0.14 pups per day (see Figure S2). The regression model predicts an average litter size at birth of 5.47. To get an estimate for adult survival, we used individuals which had known birth and death date and which lived longer than 15 days for the analysis that were not necessarily part of the pup survival analysis.

Both pup and adult survival were integrated to estimate the whole survival curve (l_x curve, probability to survive into age class x). Average loss in litter size during the first 15 days (pups survival) was used to estimate the original number of newborns giving rise to the 267 individuals available for adult survival. The resulting Kaplan-Meier plot using a bin size of 50 days is shown in Figure S3A. Average life expectancy was 196 days.

Fertility. In order to get reliable estimates of net reproductive rate (R_0) and generation time (γ), only the 267 individuals where information about survival were known were included in the fertility analysis. Parentage information and the birth dates of the parents and the offspring

was available in 1258 cases. Figure S3B shows the average number of offspring produced in the different age classes (m_x curve).

Net reproductive rate R_0. The average amount of offspring produced by each individual (net reproductive rate R_0) can be determined from the survival and fertility curves and is simply $\sum l_x m_x$. Using this formula, we get a total R_0 of 1.12. This roughly corresponds to an intrinsic growth rate of $r = 0.16$ per year. This increase could be compensated by emigration (since population size is observed to be more or less constant).

Generation time. If survivorship l_x is defined as the probability to survive from birth to age class x and fecundity m_x is the number of offspring born to the parent of age class x generation time is

$$\gamma = \frac{\sum x l_x m_x}{\sum l_x m_x}.$$

γ can therefore be seen as a weighted mean at which any individual in the population had offspring, just as defined above. Using these survival and fertility distributions and an age-class-size of 50 days, total generation time was $\gamma = 263$ days.

N_e — **Effective Population Size.** In 2004, an average of 46 sexually mature adults were present in the population (Lindholm, unpublished). For our model, we assume an average population size of 50.

τ — **Transmission ratio distortion.** Among the twelve founder individuals, four were heterozygote t carriers. Absence of t/t homozygotes among all 2190 pups support the idea that there is only one version of the t haplotype occurring in the study population. Transmission ratio distortion (TRD) was estimated as 0.90 in controlled laboratory crosses using captured mice from the study population and their lab-born descendants (Lindholm, unpublished). This value strongly deviates from Mendelian expectations ($\chi^2_1 = 110.41, P < 0.001$). t frequency over the whole time period among all pups was $\bar{p}_t = 0.11$.

s_1, s_2 — **Differences in survival between $+/+$ and $+/t$ individuals.** Survival depending on ge-

netic background was only analysed for individuals that lived longer than weaning (age of 20 days, Koenig and Markl [1987]). Pup viability was excluded for reasons of data quality. For 187 males and 171 females living longer than 20 days, information about the date of birth and death (i.e. longevity) were available. Further, information about reproductive activity (i.e. age at last reproduction) was used for an additional 114 females and 103 males as censored data. To determine possible differences in survival dependent on the genotype, we performed a Cox proportional hazard model (see Figure 2). The model did not find significant differences in longevity for males ($n = 185$, $\exp(\beta)$=1.26, $P = 0.31$). For females however, survival seems to be strongly influenced by the genetic background ($n = 174$, $\exp(\beta)$=2.46, $P < 0.01$). Both models met the proportional hazards assumption.

Knowing baseline pup survival (see Text S2) and the estimates for survival from the Cox model (see Figure 2), we can calculate the fraction of individuals still present at the typical time of reproduction (i.e. generation time γ). Doing so leaves us with 16.87% of all born $+/+$ females and 21.97% $+/t$ females (resulting in $s_1 = -0.30$). On the male side, 9.98% $+/+$ and 12.2% $+/t$ males remain at generation time γ. Since this difference was not significant, it was not included into the model, thus $s_2 = 0$.

Figure S1 — Inbreeding.

Figure S2 — Pup survival.

Figure S3 — Overall survival and fertility curves.

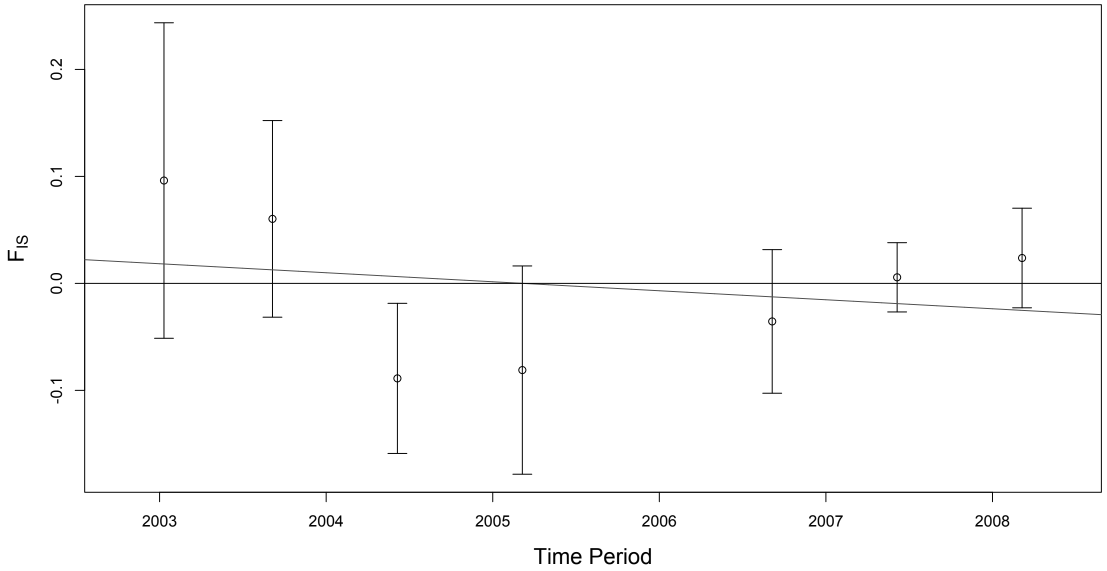

Figure 1. S4 — F_{IS} values (deviations from Hardy-Weinberg predictions) with standard deviations for 9 month time periods (identical the ones used in the model) with standard deviations. The grey line shows the linear regression line ($n = 147$, $P = 0.07$, $r = 0.023$) and the grey line the Hardy-Weinberg predictions.

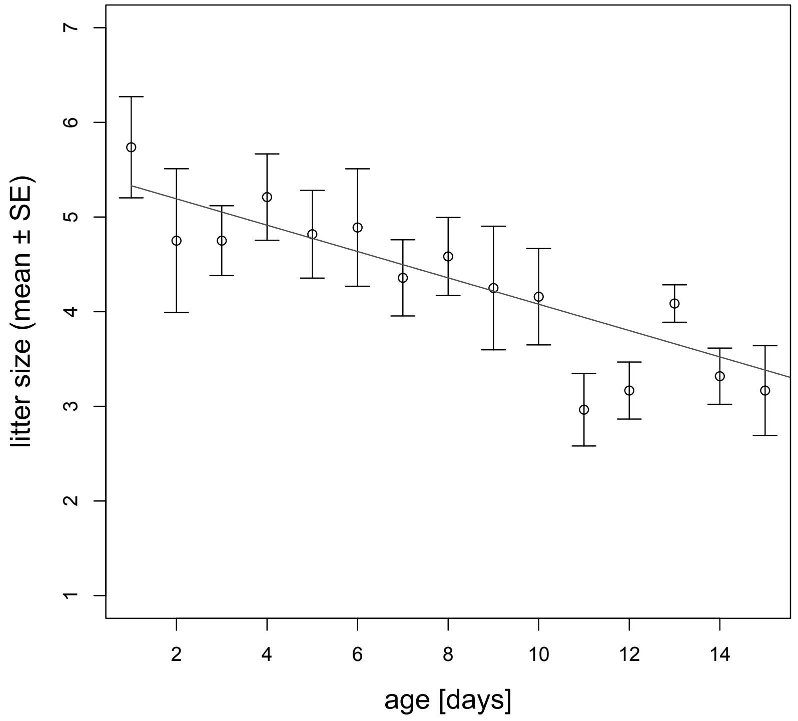

Figure 2. S2 — Average litter size with standard errors dependent on age of the litter with linear regression prediction (grey line, $n = 434$, $p < 0.001$, $r = 0.07$).

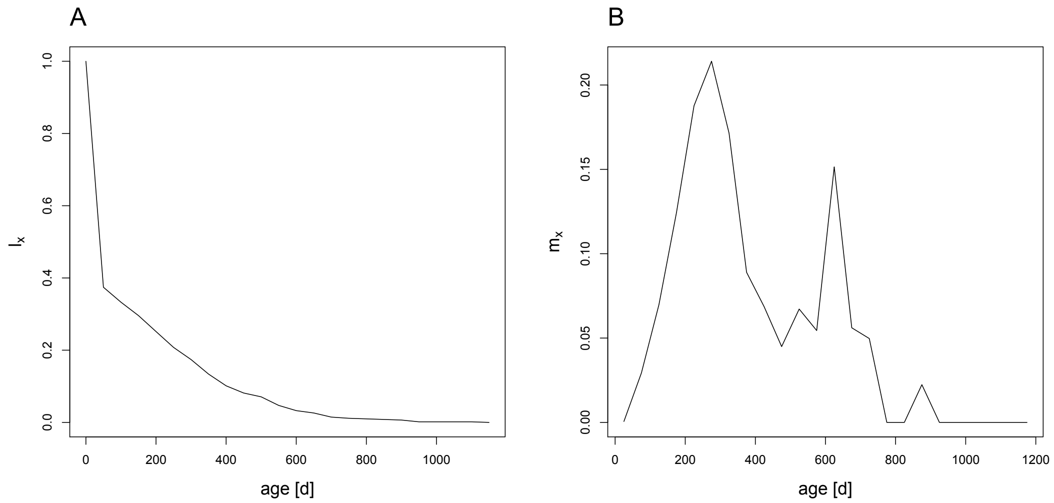

Figure 3. S3 — (A) Survival curves (Kaplan-Meier estimates) for males and females based on an age-class-size of 50 days. (B) Average number of offspring produced in these age classes. The three maxima seem to correspond to different years, suggesting seasonality in fertility.
